# The space-time continuum in neurological disorders of the autophagosome-lysosome fusion machinery

**DOI:** 10.1080/27694127.2025.2560903

**Published:** 2025-10-12

**Authors:** Hormos Salimi Dafsari, Juliane Schuler, Emil Schober, Birk Möller, Adam Antebi, Manolis Fanto, Heinz Jungbluth

**Affiliations:** a Department of Pediatrics and Center for Rare Diseases, Faculty of Medicine and University Hospital Cologne, University of Cologne, Cologne, Germany; b Max-Planck-Institute for Biology of Ageing, Cologne Excellence Cluster on Cellular Stress Responses in Aging Associated Diseases (CECAD), Cologne, Germany; c Department of Basic & Clinical Neurosciences, Institute of Psychiatry, Psychology & Neuroscience, King’s College London, London, UK; d Department of Paediatric Neurology, Neuromuscular Service, Evelina London Children’s Hospital, Guy’s and St Thomas’ Hospital NHS Foundation Trust, London, UK; e Randall Centre for Cell and Molecular Biophysics, Muscle Signaling Section, Faculty of Life Sciences and Medicine (FoLSM), King’s College London, London, UK

**Keywords:** Autophagy, autophagosome-lysosome fusion machinery, neurodevelopmental disorders, neurodegenerative disorders, aging

## Abstract

Autophagy is a highly conserved cellular pathway for the degradation and recycling of defective intracellular cargo and plays a vital role in the homeostasis of post-mitotic tissues, particularly the nervous system. Autophagosome-lysosome fusion represents the final critical step in macroautophagy with a tightly regulated process mediated by a complex molecular machinery of tethering vesicles for degradation. Since the first reports of human autophagy disorders, the scientific and clinical focus condensed on severe phenotypes with biallelic-truncating genotypes as monogenic models of near-complete autophagy perturbation. Recent reports suggest a much wider disease spectrum with defective autophagy, ranging from neurodevelopmental disorders to neurodegenerative phenotypes with later manifestation due to “milder” genotypes, including Alzheimer’s disease (AD), Parkinson’s disease (PD), and Amyotrophic Lateral Sclerosis-Frontotemporal Dementia (ALS-FTD). In addition, recent evidence identified molecular connections between physiological autophagy regulation during normal aging and pathophysiological hallmarks of aging-related disorders. These translational observations led to a more comprehensive understanding of autophagy at health and disease, in particular: 1) genetic location and allelism of pathogenic variants (“genomic space”); 2) protein-protein interaction in functional protein complexes (“proteomic space”); 3) metabolic autophagic flux with positive and negative regulators (“metabolomic space”); 4) age-related phenotypic progression over time. Here, we review the autophagosome-lysosome fusion machinery as a key structure both on the molecular level and with regards to the pathogenesis of the autophagy-related disease spectrum. We highlight the clinicopathological signature of disorders in the autophagosome-lysosome fusion machinery, in particular features warranting awareness from clinicians and geneticists to inform adequate diagnosis, surveillance, and patient guidance.

## Introduction

Autophagy is a fundamentally important and highly conserved intracellular pathway that involves the digestion and recycling of intracellular material through a highly specialized molecular machinery for maintaining cellular homeostasis^[1]^.

Autophagy is characterized by different forms: a) macroautophagy characterized by the delivery of intracellular components through double-membraned vesicles (or autophagosomes) to the lysosome; b) microautophagy defined by direct substrate engulfment by the lysosomal membrane, and c) chaperone-mediated autophagy characterized by chaperone-mediated recognition of specific protein motifs for delivery to and direct import to lysosomes via LAMP2A^[2]^. All forms of autophagy converge in the ultimate degradation of cargos within the acidic environment of the lysosome.

We focus this review on macroautophagy (hereafter simply called “autophagy”) with its two modes: (1) Bulk autophagy that occurs mainly in response to stressors such as nutrient starvation, and (2) selective autophagy that is focussed on specific cargos such as defective organelles (e.g. mitophagy), nuclear lamina (nucleophagy), pathogens (xenophagy), or the endoplasmatic reticulum (ER-phagy), and may tag its target by ubiquitination to ultimately merge with lysosomes^[3,4]^.

The autophagy signaling pathway consists of different steps that range from initiation and nucleation of a phagophore and its formation into an autophagosome, to its fusion with the lysosome for final degradation and recycling of the cargo. The generation of autophagosomes is initiated at multiple sites throughout the cytoplasm and begins with the phagophore formation and biogenesis of autophagosomes, the double-membraned vesicles that sequester cellular cargo targeted for degradation^[5]^. Potential sites of vesicle origin are at the ER, plasma membrane, Golgi complex, or mitochondria^[6]^. The phagophore surrounds the cargo and forms the double-membraned autophagosome, while molecular complexes like the ULK1 complex and PI3K (phosphoinositide 3-kinase regulatory subunit type 3) complex interact after upstream regulation by mTORC1 or AMPK. The PI3K complex has an essential role in phagophore nucleation, consisting of core proteins, including beclin 1, ATG14, and Vps34 among others^[7]^. The phagophore elongation and closure to autophagosomes is guided by interaction of the PI3K complex, ATG9, and the ATG8 lipidation system, additionally ensuring cargo specificity through adaptor proteins such as p62 and the ATG5-ATG12-ATG16L1 complex^[6]^.

Once autophagosomes are fully formed, they are transported to the lysosome for a fusion process resulting into autophagolysosomes to expose the cargo to the acidic lumen and resident lysosomal hydrolases for degradation. This terminal fusion process is one of the most critical steps in autophagy and orchestrated by a network of molecular players, including proteins of the RAB (Ras-associated binding) and ARF (ADP-ribosylation factor) small GTPase families, working as molecular identifiers. These proteins are determining the fate of an organelle by recruiting effector proteins in their active GTP bound state^[8]^. In addition, key factors in this fusion process include tethering factors (EPG5, ectopic p-granules autophagy protein homolog 5) and molecular complexes, such as the HOPS (homotypic fusion and protein sorting) complex, SNAREs (soluble NSF attachment protein receptors), motor proteins, adaptor proteins, phospholipids, and central regulatory proteins such as RUBCN (RUN Domain Beclin-1-Interacting And Cysteine-Rich Domain-Containing Protein)^[9]^. In the course of this review, we aim to describe their functions and molecular mechanisms in detail.

The efficiency of autophagosome-lysosome fusion declines with age due to a combination of molecular and structural changes^[10]^. These include decreased RAB7 activity, which limits the recruitment of fusion machinery^[11]^; reduced stability and expression of HOPS complex components which impair tethering^[12]^; and increased levels of RUBCN, a negative regulator, that inhibits fusion^[13]^. There is also an age-related deterioration of lysosomal function, characterized by reduced acidification, enzymatic inefficiency, and the accumulation of lipofuscin which physically hinders autophagic processes^[14,15]^. Together, these changes may contribute to the accumulation of damaged proteins and organelles, exacerbating cellular dysfunction and forming the basis for neurodegenerative diseases.

**Figure 1.**
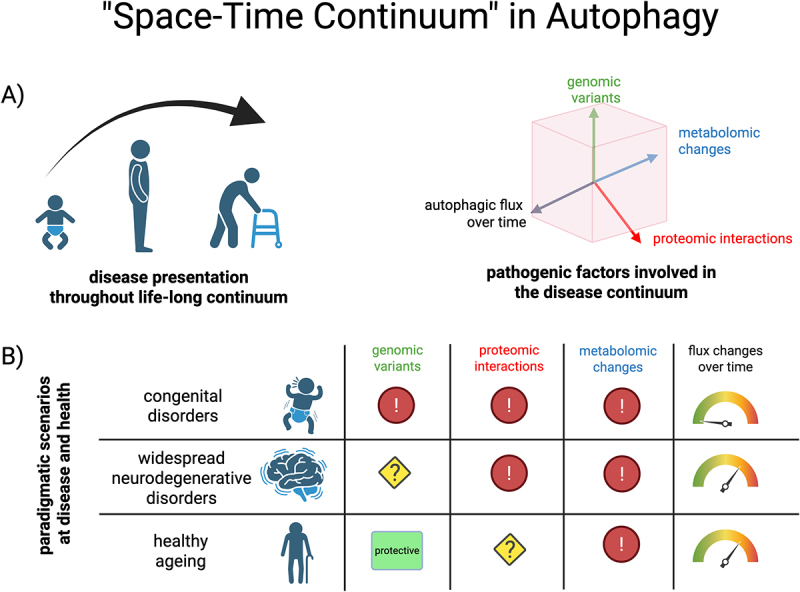
Overview on the “space-time continuum” in autophagy in health and disease. A) Health throughout the lifetime (left) depends on 1) genetic location and allelism of pathogenic variant(s) (“genomic space”); 2) the protein-protein interaction in the greater context of protein complexes (“proteomic space”); 3) autophagic flux, in particular in relation to positive and negative regulators (“metabolomic space”); 4) lastly, autophagy flux changes over the lifespan (“time”). B) Examples of dysregulations of these factors in congenital disorders, more common neurodegenerative disorders and healthy aging. Congenital disorders of autophagy present with early onset due to highly pathogenic genomic variants that cause dysfunctional protein-protein interactions and metabolic dysregulations in autophagic flux. More common neurodegenerative diseases such as parkinsonism with potential genetic susceptibility due to variants in autophagy genes present at later stages of life with dysfunctional protein-protein interactions and dysregulated autophagic flux. Healthy aging is associated with protective variants in autophagy genes and unclear proteomic interactions, but metabolic changes in autophagic flux are known to at least occur at late stages in adulthood. Created with Biorender.

In the following review, we suggest a framework of an autophagy-related “space-time continuum” characterized by the following factors (Figure 1): 1) genetic location and allelism of pathogenic variant(s) (“genomic space”); 2) the protein-protein interaction in the greater context of protein complexes (“proteomic space”); 3) autophagic flux, particularly in relation to positive and negative autophagy regulators (“metabolomic space”). Finally, 4) the autophagic flux decreases with age, even under healthy conditions, another (“time”) variable in this continuum. We will outline the key features of ultra-rare paradigmatic early-onset Mendelian disorders of different components of the autophagosome-lysosome fusion machinery, and highlight its role in physiological aging and more common late-onset neurodegenerative disorders. Finally, we will provide an outlook on potential therapeutic strategies focussing on the autophagosome-lysosome fusion machinery.

## Molecular mechanisms in canonical autophagy

**Figure 2.**
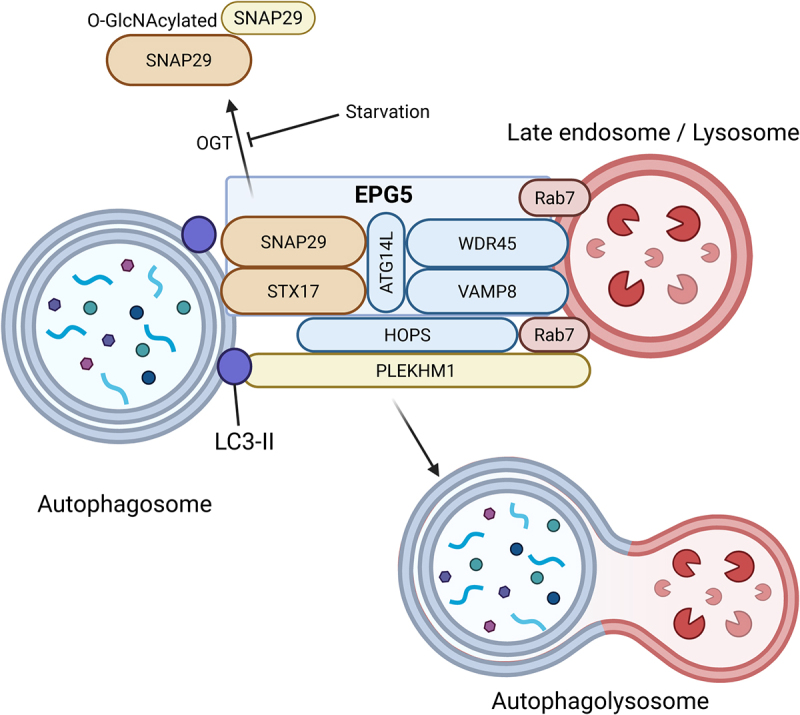
The autophagosome-lysosome fusion machinery. The fusion between autophagosomes and lysosomes in the final step of the autophagy pathway is a complex process that involves an intricate molecular machinery. In addition to EPG5, other proteins interacting within the autophagosome-lysosome fusion machinery have already been implicated in neurodevelopmental and neurological conditions or are candidates for as yet unresolved conditions. EPG5 = Ectopic P-Granules 5 Autophagy Tethering Factor; SNAP29 = Synaptosome Associated Protein 29; STX17 = Syntaxin 17; ATG14L = Autophagy Related 14; VAMP8 = Vesicle Associated Membrane Protein 8; Rab7 = Ras-related protein Rab-7a; HOPS =homotypic fusion and protein sorting-tethering complex; PLEKHM1 = Pleckstrin Homology And RUN Domain Containing M1; LC3-II = LC3-phosphatidylethanolamine conjugate.

Autophagosome-lysosome fusion is a tightly coordinated process that ensures the effective degradation of cytoplasmic cargo. Autophagosome-lysosome fusion involves multiple sequential steps, including initiation and biogenesis of the phagophore, autophagosome maturation, transport to and fusion with the lysosome and, finally, degradation and recycling of cargo in the autophagolysosome^[2]^. The primary molecular machinery governing this process consists of small GTPases (RAB7, ARL8), tethering factors (HOPS, EPG5), SNARE proteins (STX17, SNAP29, VAMP8), and motor proteins that facilitate vesicular trafficking along the cytoskeleton. Figure 2 illustrates the key proteins involved in autophagosome-lysosome fusion.

The core autophagy process starts with the assembly of the ULK1 complex, leading to its translocation from the cytoplasm to the endoplasmic reticulum (ER)^[16,17]^. The ULK1 complex includes the serine/threonine kinase ULK1 (unc-51 like autophagy-activating kinase 1), a FIP200 (FAK family kinase interacting protein of 200 kDa) scaffold, and the core autophagy proteins ATG101 and ATG13, which dimerize through HORMA (Hop/Rev7/Mad2) domains^[18]^. ULK1 plays a key role in the initiation of autophagy^[19]^. The regulation of the ULK1 complex is promoted by two factors: 1) Mechanistic target of rapamycin complex 1 (mTORC1), a central regulator of cellular metabolism, inhibits autophagy by inactivating ULK1 and ATG13 and 2) the AMP-activated protein kinase (AMPK) which registers low cellular energy levels and is activated by high cellular AMP concentrations^[18,20]^.

mTORC1 plays a key role in regulating the balance between cellular growth and catabolism and is driven to the lysosome by amino acids, glucose, and lipids (under nutrient-rich conditions), where its kinase function is active^[21]^. Under the conditions of nutrient deprivation, mTORC1 is inactivated and dissociated from the lysosome, resulting in partial dephosphorylation of ULK1 and ATG13 in the cytoplasm, which drives assembly and activation of ULK1 complex^[21]^. Under nutrient-rich conditions, the partially dephosphorylated ATG13 facilitates the interaction between phosphorylated ULK1 and FIP200 which induces the formation of the phagophore and the recruitment of other autophagic proteins^[17]^.

The AMP-activated protein kinase (AMPK) is a heterotrimeric complex composed of three subunits: one catalytic (α) and two regulatory (β and γ). Its kinase activity is regulated by phosphorylation at threonine 172 on the α-subunit as a sensor of cellular energy status by detecting the increased AMP/ATP ratio. During energy stress conditions such as starvation, ATP levels decrease significantly, leading to an accumulation of ADP and AMP. In response, AMPK activates catabolic processes including autophagy, which in turn results into degradation products that are recycled for energy production. AMPK inhibits mTORC1 activity by phosphorylating upstream regulators of the mTOR pathway. Simultaneously, it activates ULK1 by phosphorylating multiple serine residues (Ser317, Ser467, Ser555, Ser574, Ser6637, and Ser777), thereby counteracting the mTORC1-mediated suppression of autophagy^[17]^.

Activation of ULK1 with its translocation to the ER leads to phosphorylation of Beclin1 and ATG14 of the PI3K complex (class III phosphatidylinositol 3-kinase complex), subsequently activating the lipid kinase activity of the Vps34 subunit in the PI3K complex. Vps34 phosphorylates phosphatidylinositol and generates PI3P (PtdIns-3,4,5-trisphosphate) at the phagophore assembly site to act as receptor for PI3P-binding effects such as WIPI2 (WD-repeat proteins interacting with phosphoinositides), in turn recruiting the ATG8/LC3 lipidation machinery to the phagophore^[18,22,23]^.

The elongation of the phagophore, is dependent on two ubiquitin-like conjugation pathways: 1) the ATG12-ATG5-ATG16L1 complex formation, which requires ATG7 and ATG10 acting as ubiquitination-activating enzyme 1 and 2 (E1 and E2) leading to the conjugation of ATG5 to ATG12 and later the dimerization of ATG16L1, which allows the association with the phagophore promoting the expansion of the membrane^[6]^. The second complex involved in the elongation is ATG7 and ATG3 acting with the ATG12-ATG5-ATG16 complex to form the lipidation machinery of ATG8. ATG7 (E1), ATG3 (E2), and ATG12-ATG5-ATG16L1 complex (a ubiquitin E3 ligase) facilitate the covalent binding of ATG8 to the lipid phosphatidylethanolamine (PE)^[6,22]^ which is then integrated in the autophagosome membrane and promotes the closure of the phagophore. There are six isoforms of ATG8-like proteins, divided into the LC3 and GABARAP subfamilies^[6]^. These subfamilies are defined by amino acid sequence similarity: While the LC3 subfamily consists of LC3A, LC3B, LC3B2, and LC3C, the GABARAP subfamily includes GABARAP, GABARAPL1, and GABARAPL2. All subfamilies have a role in autophagosomal biogenesis: The ATG8/LC3 subfamily plays a more prominent role at earlier stages, by mediating the elongation of the phagophore, while GABARAPs are suggested to mediate the fusion step of autophagosomes and lysosomes by regulating the lipid composition^[8]^. The lipidated, PE-conjugated form of ATG8/LC3 is called LC3-II and can be used as a marker for autophagic activity as it is also degraded by lysosomal proteases after the fusion^[6,24]^.

After successful formation of autophagosomes, their fusion with lysosomes is dependent on their trafficking to lysosomes with the aid of microtubules^[25]^. Once autophagosomes are in proximity to lysosomes, tethering factors stabilize their interaction: The HOPS complex, a six-subunit tethering factor, plays a central role in this step. HOPS binds RAB7 on the autophagosome and lysosome, facilitating docking through its interactions with SNARE proteins. Additionally, EPG5 acts as an alternative tether, interacting with STX17, LC3, and lysosomal membrane proteins such as LAMP2 (lysosome-associated protein 2), ensuring that autophagosomes are properly aligned for membrane merging. The final fusion step is mediated by the formation of a trans-SNARE complex. Syntaxin-17 (STX17) localizes to autophagosomal membranes, where it interacts with the cytosolic Qbc-SNARE SNAP29 and the lysosomal R-SNARE VAMP8 to drive membrane fusion^[26]^. In addition, mature autophagosomes can fuse with endosomes to form amphisomes^[16]^.

At the final step of autophagy, newly supplied nutrients activate mTORC1-mediated autophagy inhibition and stimulate autophagic lysosome reformation (ALR). To terminate autophagy, ALR is regulated by several cellular proteins, such as kinesin, dyneins, clathrin-mediated endocytosis proteins, the ubiquitin E3 ligase Cullin 3-Kelch-like protein 20 (KLHL20), and lysosomal efflux permeases^[16]^.

Bulk autophagy is activated by nutrient deprivation or various energy stress conditions to support cellular survival by recycling cellular components. Selective autophagy on the other hand can occur in the absence of starvation and reacts to specific stressors like mitochondrial damage, oxidative stress, or pathogens, to ensure cellular homeostasis and maintenance by removing defective organelles and macromolecules^[19]^. Cargo recognition in selective autophagy is mediated by specific receptor proteins that bind either directly or via ubiquitin while simultaneously interacting with the expanding isolated membrane through ATG8-family proteins^[27]^. In addition, these cargo receptors recruit components of the autophagy machinery to facilitate the localized initiation and formation of the autophagosome^[27]^. The autophagy adaptor protein p62/SQSTM1 is involved in several forms of selective autophagy alongside other proteins: Bcl2-L-13, Nix/BNIP3L, NDP52, and OPTN are involved in the degradation of damaged mitochondria (“mitophagy”), while NDP52 and OPTN are also involved in the selective clearance of intracellular pathogens (“xenophagy”)^[2]^. TRIM (tripartite motif-containing) superfamily proteins are also involved in the regulation of selective autophagy for immunity and cellular development^[28]^. Their N-terminus consists of (1) a RING finger domain which confers E3 ubiquitin ligase activity and mediates ubiquitylation, (2) one or two B-boxes, whose function remains incompletely understood, (3) and a coiled-coil-domain (CCD) which facilitates the formation of high-molecular-weight complexes through interactions either among TRIM-family members or other proteins^[28]^. Several TRIM proteins, such as TRIM5, TRIM20, or TRIM21, serve as cargo receptors in selective autophagy and as scaffolds for the assembly and activation of the core autophagy machinery, including ULK1 complex and the PI3K complex^[29]^.

One intriguing aspect is that most proteins involved in autophagosome-lysosome fusion are not specific to autophagy, but also part of a wider network involved in organelle and vesicle remodeling and fusion. This process, where a single polypeptide chain performs multiple physiologically relevant functions is also called “protein moonlighting”^[30]^. One example for this concept with regard to a protein serving at multiple steps in the autophagy pathway is found with the WIPI2 proteins: WIPI2 is a key regulator of ATG8 lipidation in the process of nonselective autophagosome biogenesis, as described earlier. Another role of WIPI2 was found in relation to mitophagy, where WIPI2 is recruited to damaged mitochondria alongside VCP upon mitophagy induction and is ultimately implicated in cell fate decisions following mitochondrial damage that involves caspase 3 and PARP1^[31]^.

Whilst the evolutionary origin of this increasingly intricate network and its purpose are currently still not fully understood, the still expanding cell biology literature on this topic suggest substantial crossover between these pathways.

### Disorders due to mutations affecting the autophagosome-lysosome fusion machinery

Human diseases associated with monogenic mutations in key autophagosome-lysosome fusion machinery genes are associated with several neurodevelopmental and neurodegenerative disorders. Table 1 summarizes selected monogenic congenital disorders of autophagy that are discussed within this review.

**Table 1.** Selected disease genes with congenital disorders of autophagy and their associated neurological disease presentations.

Disease genes	Associated neurological disease presentations
*DYNC1H1*	neuromuscular and neurodevelopmental disorders, progressive neurodegenerative diseases including spastic paraplegia and parkinsonism
*RAB7A*	neuropathy
*WDR45*	β-propeller protein-associated neurodegeneration (BPAN) including neurodevelopmental disorder, dystonia, parkinsonism, epilepsy
*SNAP25B*	congenital myasthenia, ataxia, neurodevelopmental disorders
*SNAP29*	Cerebral dysgenesis–neuropathy–ichthyosis–keratoderma (CEDNIK) syndrome
*VPS41*	spinocerebellar ataxia, dystonia, parkinsonism
*VPS16*	dystonia
*EPG5*	neurodevelopmental disorders, epilepsy, parkinsonism; Vici syndrome
*RUBCN*	ataxia

While the clinical field has long considered neurological disorders during development as mostly distinct from degenerative diseases of later onset, recent efforts in large-scale genome sequencing have identified individuals with a spectrum of diseases with either infant-onset manifestations (including Dravet syndrome and autism) or adult-onset movement disorders (including Huntingon’s disease)^[32–38]^. These clinical observations have spurred molecular biological studies into these disorders that led to the identification of shared defective signaling pathways.

Significant advances in molecular biological research have addressed pathogenic mechanisms in the manifestation of both neurodevelopmental and neurodegenerative diseases. Autophagy is involved in several steps of neurodevelopment; however, its effects may either promote or inhibit neuronal development and differentiation, depending on the specific developmental stage and cell type^[39]^. Recent studies suggest that the neurodevelopmental abnormalities observed with the ablation of autophagy-related genes may arise from their non-canonical functions rather than from a direct disruption of core or selective autophagic processes. For instance, the tethering factor EPG5 has been implicated in both human and murine stem cell development due to its non-canonical interaction with USP8^[40]^, and has only recently been implicated in age-related neurodegenerative processes due to defects in mitophagy^[41,42]^ and the onset of seizures^[43]^. The peculiar age distribution between infantile developmental and adolescent neurodegenerative disease presentations in *EPG5*-related disorders may serve as a paradigm for the dichotomy of the underlying pathogenesis of molecular biological processes related to defective autophagy. However, several autophagy-related disease genes may not present with this age gap between developmental and degenerative disease manifestations, and instead potentially show a more succinct overlay of both during early periods of life. As an example for the latter overlay, pathogenic variants in *WDFY3* (WD repeat and FYVE domain containing 3) were identified in macrocephaly, neurodevelopmental delay, seizures, and autism spectrum disorder (ASD) which may prompt the clinical impression of a primarily neurodevelopmental disorder^[39]^. While the non-canonical role of WDFY3 in interaction with the Wnt signaling pathway may be a driving force in perturbed brain development, recent studies in cellular and animal models have shown shortened lifespan and neurodegeneration with dysfunctional mitophagy^[44,45]^. It is unclear whether an early *in utero* or infantile onset of neurodegeneration on top of the neurodevelopmental disorder drives the manifestation and severity of seizures in human WDFY3 dysfunction or other congenital disorders of autophagy.

Here, we focus on the autophagosome-lysosome fusion machinery as a fulcrum in human disease with its age-dependent roles in developmental and degenerative processes. Autophagosomes are found throughout the cytoplasm, while lysosomes are predominantly located in the perinuclear region. Any fusion event relies on the shuttling and meeting of both and may require long-range trafficking mechanisms particularly in neurons in which some axons may exceed 1 m in length^[46]^. The trafficking mechanisms mainly include cytoskeletal structures, motor proteins and other adaptor or receptor proteins^[25]^. Autophagosomes move along cytoskeletal structures such as microfilaments (actin filaments) and microtubules with the help of molecular motors. Actin filaments form a dynamic network that facilitates the trafficking of organelles to the autophagosome, a process regulated by the nucleation promoting factors ARP2/3 and the actin-capping protein CapZ that as a scaffold support the directional transport of the autophagosome, as well as membrane expansion and fusion with the lysosome^[25]^. Molecular motors involved in autophagosomal trafficking include myosin, which is mostly supporting the formation, expansion and maturation of the autophosome by transporting vesicles and membrane components, and microtubule-based motors such as dynein, which is directly involved in the trafficking of the autophagosome to the lysosome^[25,47]^. The two groups of motor proteins are dynein for retrograde and kinesin for anterograde trafficking. As autophagosomes mainly move toward the nucleus to meet the lysosome in the perinuclear region, retrograde movements facilitated by dynein play an important role in the trafficking of autophagosomes^[25]^. Another key component in autophagosome trafficking is LC3/ATG8 that links the autophagosome to microtubules. In neuronal cells, LC3/ATG8 further contributes to directional regulation through its interaction with the scaffolding protein JIP1 which suppresses kinesin-driven anterograde trafficking and simultaneously facilitates the dynein-dependent retrograde trafficking^[25]^. As the output of autophagosome-lysosome fusion depends on the efficiency of this long-range transport mechanism, trafficking proteins may also critically regulate autophagic flux. For this reason, the narrative in this review includes retrograde trafficking events before we proceed to the autophagosome-lysosome fusion machinery itself.

### DYNC1H1: retrograde trafficking

The cytoplasmic dynein 1 heavy chain 1 (DYNC1H1) homodimer is the core motor subunit of the cytoplasmic dynein 1 motor complex, which is found in all eukaryotic cells and functions as the primary retrograde transport effector for vesicles, organelles, and protein aggregates along microtubules^[48–51]^. Driven by the DYNC1H1 motor, the primary active retrograde transport mechanism in neurons is provided by the cytoplasmic dynein 1 protein^[51–53]^.

The interaction of DYNC1H1 with autophagy regulators is multifaceted, integrating various signaling pathways and proteins that coordinate vesicle transport and fusion. The FTS-Hook-FHIP ( = FHF) cargo adaptor complex is required to connect dynein to a specific cargo. Early endosomes are marked by Rab5, the master regulator of formation, homotypic fusion and motility of the early endosome^[54,55]^. The interaction of FHIP1B and Hook 1 or 3 forms the FHF complex, which connects dynein to the early endosome via Rab5B. The connection of dynein with the early endosome enables the initialization of active retrograde transport^[56,57]^. Additionally, dynein interacts with Rab7, a master regulator of autophagosome-lysosome fusion and marker of the late endosome. Rab7-regulated, dynein-dependent retrograde transport is a key link for the maturation and degradation of dendritic endosomes^[55]^. Furthermore, Rab7 facilitates the recruitment of the HOPS complex, which stabilizes tethering of autophagosomes to lysosomes. The dynein adaptor protein JIP4 (c-Jun N-terminal kinase-interacting protein 4) is required for RAB7-driven retrograde transport, linking DYNC1H1 to autophagosome positioning^[58]^. DYNC1H1 also engages with SNARE proteins, including Syntaxin-17 and SNAP29, ensuring that properly positioned autophagosomes can undergo efficient membrane fusion^[53]^.

Disruptions in dynein function impair autophagosome trafficking, leading to the accumulation of immature autophagic vesicles, defective cargo clearance, and exacerbation of cellular stress, particularly in neurons, which are highly reliant on the efficient organelle transport for maintaining homeostasis, where it has been demonstrated that the dysfunction results in neuritic swelling and intracellular β-amyloid accumulation^[59]^. Due to the loss of dynein function, the spatial coordination of these interactions is disrupted, resulting in autophagic vesicles stuck in distal neuronal compartments, defective endosome maturation and impaired autophagic flux, contributing to synaptic dysfunction and neurodegeneration^[59,60]^.

Pathogenic variants in *DYNC1H1* are linked to a wide clinical spectrum referred to as *DYNC1H1*-related disorders, which can be subdivided into:

1. *DYNC1H1*-neuromuscular disorder (NMD) characterized by exclusive involvement of the peripheral nervous system and a solely neuromuscular phenotype. The NMD phenotype includes spinal muscular atrophy, lower extremity-predominant 1 (SMA-LED1; MIM#158600) and Charcot-Marie-Tooth disease, axonal, type 2O (CMT20; MIM#614228).
2. *DYNC1H1*-neurodevelopmental disorder (NDD) characterized by concomitant CNS involvement, which may additionally include symptoms from the *DYNC1H1*-NMD spectrum. The NDD phenotype includes malformations of cortical development (MCD) as well as autosomal dominant mental retardation 13 (MRD13; MIM#614563)^[61–63]^.

Recent insights into pathogenic *DYNC1H1* variants have linked this disease to a range of age-dependent phenotypes^[64]^. Early in life, the *DYNC1H1*-related disease spectrum is mainly defined by NMD and NDD phenotypes. NMD presentations are associated with mutations in the DYNC1H1 dimerization domain that lead to impaired retrograde axonal transport, progressive motor neuron degeneration with predominantly lower limb muscle loss and weakness, variably associated with skeletal malformations, and delayed motor milestones^[63]^. NDD presentations are characterized by motor and sensory neuropathy, global developmental delay, brain malformations, epilepsy, and movement disorders due to defective autophagosome transport, accumulation of misfolded proteins, and neuronal migration defects^[62,65,66]^. These clinical manifestations have been described as part of a life-long continuum and with age-related disease progression, which can be exacerbated by viral infections due to an infection-related progressive impairment of intracellular trafficking^[64]^.

In later life, transport protein deficits, like *DYNC1H1*-associated dysfunction, are implicated in neurodegenerative diseases, including Alzheimer’s, Huntington’s and Parkinson’s disease, where defective autophagy may exacerbate the accumulation of tau aggregates and α-synuclein inclusions, respectively^[67–69]^. In particular, animal models have also shown that dynein dysfunction increases the toxicity of Huntington’s disease-causing mutations^[60,70,71]^. Age-related decline in dynein-mediated transport, compounded by lysosomal dysfunction, also contribute to progressive neuronal vulnerability, leading to cognitive impairment and motor deficits^[64]^.

Emerging evidence suggests that the age-related decline in DYNC1H1 function contributes to impaired mitophagy, the selective clearance of damaged mitochondria, further linking dynein dysfunction to oxidative stress and cellular aging^[72]^. In conditions such as hereditary spastic paraplegia (HSP), pathogenic variants in *DYNC1H1* cause progressive corticospinal tract degeneration, reflecting a failure of autophagosomal transport in upper motor neurons^[73]^. As a result, impaired *DYNC1H1*-dependent autophagy represents a unifying mechanism underlying a diverse set of neurological disorders, with increasing severity and penetrance across the lifespan. Targeting dynein activity or enhancing its interaction with autophagy regulators may provide a therapeutic avenue for treating *DYNC1H1*-related disorders and mitigating age-associated neurodegeneration in otherwise healthy populations.

### Rab-family proteins: the master regulators

Rabs are a large family of Ras-like GTPases, which function as switch molecules by changing between a GDP-bound inactive (cytosolic) state and a GTP-bound active (membrane-associated) state^[74]^. The protein superfamily is subdivided into the Arf/Arl, Rho, Ras-, Ran-, and Rab-families, which also consist of several members^[75]^. Rabs are regulated by different upstream regulators such as Rab escort proteins (REPs), which provide the lipid modification for a stable and targeted attachment onto the membrane surface. The switch from GDP- to GTP-bound states is regulated by Guanine nucleotide exchange factors (GEFs; facilitating GDP- to GTP-bound) and GTPase activation proteins (GAPs; facilitating GTP- to GDP-bound). Other key proteins in this signaling pathway include GDI displacement factors (GDFs) or GDP dissociation inhibitors (GDIs) that execute the attachment of Rab to the membrane^[75]^. Various Rab small GTPase proteins are involved in the fusion between autophagosome and lysosome such as Rab5 and Rab7^[8]^. Rab7, in its GTP-bound form, is known to interact with various effectors (motors, adaptors and tethering factors) to regulate several downstream functions such as membrane fusion upon interaction with Rab effector proteins^[75]^. The GTP-bound form of Rab7 localizes to the autophagosomal membrane, recruiting motor proteins and tethering proteins such as the HOPS complex, specific members of the SNARE family, and EPG5 to facilitate transfer of the cargo to the lysosome and tethering with lysosomes^[8,76,77]^. The HOPS complex contains the proteins VPS39 (GEF) and TBC1D15 (GAP) involved in the trafficking of cargo and fusion of autophagosome with lysosome^[75]^. Other downstream effectors include RILP (Rab7-interacting lysosomal protein) and FYCO1 (Fab1-YotB-Vac1p-EEA1) or MON1 (Monensin sensitivity protein 1) and CCZ1 (caffeine, calcium and Zinc1) which are also involved in core autophagy processes^[75]^. Especially the late steps in the fusion process require Rab7 as well as the maturation from early to late endosome, endosome motility, perinuclear clustering of late endosomes, and mitophagosome formation^[55,78]^.

In the first phase of autophagosome-lysosome fusion, these vesicles are prepared for the contact: 1) the HOPS complex converts Rab7 into its GTP-containing form and 2) the cis-SNARE complexes, localized on the same membrane, are disassembled to generate active, fusion-competent monomeric SNARE proteins^[75]^. During tethering, the HOPS complex interacts with Rab7-GTP, while unpaired monomeric SNAREs assemble into trans-SNARE complexes bridging opposing membranes. The final fusion of the membrane is facilitated by the SNARE complex which is discussed below^[75]^.

This complex system highlights the important role that Rab7 plays in the regulation of endo-lysosomal and autophagosomal membrane trafficking. Post-translational modifications, such as phosphorylation and S-nitrosylation, modulate Rab7 activity, ensuring precise regulation of fusion events. In addition, recent insights emphasize the critical role of Rab7 in mitophagosome formation, particularly within the PINK1-Parkin-mediated mitophagy pathway^[79]^. Beyond its well-established role in autophagosome-lysosome fusion, Rab7 is actively recruited to damaged mitochondria via its guanine nucleotide exchange factors (GEFs), such as the MON1-CCZ1 complex, which facilitate the transition of GDP-bound inactive Rab7 to its active GTP-bound state at the mitochondrial surface. This recruitment process is tightly regulated by mitochondrial stress signals, including depolarization-induced PINK1 accumulation and Parkin activation, leading to enhanced Rab7 localization to mitophagic vesicles. As indicated before, other Rab proteins are involved in autolysosome or amphisome fusion such as Rab1, Rab2, Rab5, or Rab14. Recent studies have indicated interactions between Rab7, Rab2 and Rab14, assembling into a complex on the lysosome or late endosome to promote autophagosome-lysosome fusion^[80]^.

As a pivotal GTPase for vesicle docking and fusion, Rab7 activity declines with age, resulting in impaired trafficking of autophagosomes from the cytosole to lysosomes^[74]^. This age-related decline is associated with inefficient clearance of damaged organelles and aggregated proteins, increasing vulnerability to neurodegeneration^[81]^. Heterozygous pathogenic variants in its gene *RAB7A* are associated with impaired autophagic flux and cause a form of neuropathy (Charcot-Marie-Tooth disease type 2B)^[82]^. There are no clear genotype-phenotype correlations sufficiently explaining how allelism contributes to disease severity. Interestingly, the *RAB7A*-associated neuropathy shares similarities with the neuropathy occasionally seen in *EPG5*-mutated patients, indicating a clinical correlate of the close molecular interactions between the two proteins. Notably, several studies suggest a modifying effect of Rab7 mutations on the progression of neurodegenerative diseases, including Alzheimer’s disease (AD) and Parkinson’s disease (PD)^[78]^.

### WDR45: scaffold in fusion

The WD40 repeat protein family functions as an essential scaffold in the phosphatidylinositol 3-phosphate (PI3P)-mediated regulation of autophagy. Mammalian cells contain four WD40 repeat-containing proteins which interact with phosphoinositides (WIPI proteins): WIPI1, WIPI2, WIPI3/WDR45B, and WIPI4/WDR45^[83]^. WIPI1 and WIPI2 play important roles in autophagosome formation by recruiting the ATG12-ATG5-ATG16L1 complex for ATG8/LC3-lipidation on the phagophore membranes, as well as in tethering phagophores with the ER by interacting with ULK1-RB1CC1/FIP200 and VAPA-VAPB^[84]^. WD repeat domain 45 (WDR45), also known as WIPI4, is a key regulator of autophagy and lysosomal homeostasis. WDR45 is highly expressed in neurons, where it facilitates autophagosome formation, maturation, and lysosomal fusion, processes that are crucial for neuronal maintenance and survival. Loss-of-function mutations in WDR45 lead to disrupted autophagic flux, lysosomal dysfunction, and accumulation of damaged proteins and organelles, predisposing affected individuals to neurodegeneration.

In the early stages of autophagy, WDR45 interacts with the autophagy-related protein ATG2, which facilitates lipid transfer for autophagosome membrane expansion. It also binds to PI3P at the phagophore, recruiting autophagy effectors such as ATG9A and ATG16L1, which regulate autophagosome elongation. Beyond its function in autophagosome biogenesis, WDR45 is increasingly recognized as a regulator of autophagosome-lysosome fusion. It interacts with RAB7, a master regulator of vesicle trafficking, and facilitates the recruitment of the HOPS complex, which stabilizes tethering between autophagosomes and lysosomes and also interacts with EPG5^[84,85]^. Studies have demonstrated that, in the absence of WDR45/WDR45B, EPG5 is mistargeted to autophagosomes, which comprises its interaction with the SNARE complex and disrupts the assembly of the fusion machinery^[84]^. The loss of WDR45 disrupts these interactions, leading to stalled autophagic vesicles, impaired lysosomal degradation, and increased neuronal vulnerability.

Pathogenic variants in *WDR45* are associated with beta-propeller protein-associated neurodegeneration (BPAN), which is characterized by impaired autophagic flux, lipid dysregulation, and iron accumulation in the brain^[42]^. BPAN follows an age-related disease trajectory, with early-onset neurodevelopmental impairment followed by adolescent or adult-onset parkinsonism and dementia. During childhood, individuals with WDR45 disorders often exhibit global developmental delay, intellectual disability, and epilepsy, reflecting the importance of autophagy in neuronal development and synaptic maintenance. In adolescence and early adulthood, patients may develop progressive motor dysfunction, including dystonia, spasticity, and parkinsonian features such as bradykinesia and rigidity, indicative of basal ganglia degeneration due to impaired autophagic clearance of damaged mitochondria and protein aggregates.

As the disease progresses into later adulthood, *WDR45*-related disorders contribute to cognitive decline and dementia, resembling features of Alzheimer’s disease (AD) and frontotemporal dementia (FTD). The accumulation of dysfunctional autophagosomes in neurons leads to increased oxidative stress, tau pathology, and synaptic dysfunction, further exacerbating neurodegeneration. Furthermore, defective mitophagy, caused by impaired WDR45-dependent recruitment of mitophagic regulators such as PINK1 and Parkin, results in persistent mitochondrial dysfunction, linking *WDR45* variants to neurodegenerative diseases such as Parkinson’s disease (PD). Age-related decline in lysosomal function further amplifies the effects of WDR45 loss, accelerating the onset of neurodegenerative symptoms.

BPAN and other *WDR45*-related disorders highlight the critical role of autophagy in maintaining neuronal health across the lifespan. Given the progressive nature of *WDR45*-associated neurodegeneration, strategies aimed at restoring autophagic flux, enhancing lysosomal function, and modulating iron homeostasis may provide therapeutic opportunities for managing these disorders and mitigating their age-dependent progression.

### SNARE proteins: the final fusion step

SNAP receptor proteins (SNARE) are involved in the fusion of intracellular membranes. They contain a C-terminal transmembrane domaine (TMD) for attaching the membrane, a conserved SNARE motif, and a variable N-terminal domain containing four antiparallel helix bundles from one of each of the Qa, Qb, Qc, and R SNARE proteins localized in opposing membranes^[86]^. SNARE proteins are classified into vesicle membrane-SNAREs (v-SNAREs), which are anchored in the membrane of the autophagosome, and target membrane-SNAREs (t-SNAREs), anchored in the endolysosome, as the targeted membrane^[16]^. They mediate the final step of autophagosome-lysosome fusion in autophagy.

Previous studies differentiate two SNARE complexes involved in bulk autophagy^87^:

1) Syntaxin-17 (STX17) localized on autophagosomes, VAMP8 localized on lysosomes, and SNAP29 forming a trans-SNARE complex, driving membrane merging. After transferring to the autophagosome by two tandem transmembrane domains containing glycine zipper-like motifs, Stx17 recruits SNAP29. SNAP29, a Q_ab_-SNARE, is devoid of a transmembrane domain and associates with the membrane of organelles, such as Golgi apparatus, endosomes, and lysosomes by interacting with other factors such as STX17^[26]^. STX17 and SNAP29 build the Q_abc_ bundle on the autophagosome, which then forms the complex with VAMP8, the R-SNARE localized on the lysosome^[26]^. SNARE protein activity is tightly regulated by accessory factors, such as Rab GTPases and tethering factors, to prevent premature or aberrant fusion. The *O*-GlcNAc-modification of SNAP29, which is reduced under starvation conditions, results in the formation of a more stable complex with STX17 and VAMP8, and also hinders the formation of the STX17-SNAP29-VAMP8 SNARE complex, possibly due to steric hindrance caused by this modification^[88]^. EPG5 plays a critical role in this autophagosome-lysosome fusion process by stabilizing the trans-SNARE complex through interactions with STX17 and SNAP29. Acetylation of STX17 controls autophagosome maturation^[89]^.

2) The second SNARE complex, YKT6-SNAP29-STX7, is recruited to autophagosomes via the N-terminal longin domain and STX7 localizes to the lysosome^[87]^. YKT6 is a R-SNARE protein, which has received increased attention in recent years, and contains a longin domain in its N-terminus and C-terminus, on which membrane targeting is dependent. YKT6 facilitates efficient membrane fusion by forming a complex with STX17 and SNAP29 on the phagosome to prime STX17. After complex formation, YKT6 is displaced by VAMP8 resulting into the STX17-SNAP-VAMP8 complex. Released YKT6 then forms the YKT6-SNAP29-STX7 complex that drives the autolysosome formation^[26,90]^. How this complex is integrated in the interactions with other regulatory factors such as the HOPS complex or EPG5 remains unclear^[90]^.

STX17 not only forms a complex with SNAP29, which is associated with bulk autophagy during starvation, but has been suggested to contribute to the STX17-SNAP47-VAMP7/8 complex which additionally mediates autophagosome-lysosome fusion in selective autophagy under non-starving conditions^[87]^. SNAP29 recruitment is blocked by O-GlycNAcylation of SNAP29 and SNAP47 binds through PI(4,5)P2 and ATG8 to the phagophore, where it remains throughout the autophagosome stage. SNAP47 is the first SNARE protein identified to contain a lipid-binding domain, enabling its recruitment via its PH domain rather through vesicle trafficking or hydrophobic membrane anchoring, as observed for SNARES such as YKT6^[87]^.

Selective mitochondrial autophagy is a PINK1/Parkin-dependent vesicle transport pathway via STX17-SNAP29-VAMP7 for the delivery of stress-induced mitochondria to the late endosome, seemingly dependent on the HOPS tethering complex^[91]^. STX17 was identified in an eukaryotic common ancestor, indicating that the removal of damaged mitochondrial content may represent one of the earliest and most essential vesicle transport routes in the cell.

SNAREs at the lysosome act in coupling with autophagosomal STX17-SNAP29. VAMP8 and VAMP7 trafficking to lysosomes was identified to be regulated by RAB21 and MTMR13. Their depletion was found to effectively reduce VAMP8 or, alternatively, VAMP7-dependent lysosomal distribution with downstream effects on autophagosome-lysosome fusion events^[26]^. VAMP7B is bound to STX17, while the lysosome-localized DIPK2A (divergent protein kinase domain 2A) can promote autophagosome-lysosome fusion^[92]^. Apart from its role in mediating autophagosome-lysosome fusion, VAMP7 is also involved in early autophagic process regulation^[93]^, again signifying protein-protein moonlighting^[30]^.

Mutations in SNARE proteins such as SNAP25 and SNAP29, or disruptions in their interaction with tethering factors like EPG5, have been implicated in neurodevelopmental disorders, likely due to their (partial) effect on autophagosome-lysosome fusion. Monoallelic variants in *SNAP25B* are associated with congenital myasthenia, ataxia, and intellectual disability^[94]^. Biallelic variants in *SNAP29* are associated with neurocutaneous syndrome characterized by cerebral dysgenesis, neuropathy, ichthyosis, and keratoderma (CEDNIK syndrome)^[95]^. Notably, heterozygous deletions in *SNAP29* were found in 22q11 syndrome with kidney defects^[96]^. Interestingly, STX17 defects may reduce autophagosome-lysosome fusion efficiency, leading to the accumulation of undigested autophagic vesicles and contributing to tau pathology in Alzheimer’s disease^[97]^.

### HOPS complex and PLEKHM1: stabilizing and facilitating tethering and fusion

The HOPS (homotypic fusion and protein sorting) complex acts downstream of Rab7 and is composed of six subunits including the vacuolar protein sorting proteins (VPS): VPS33A, VPS16, VPS11, VPS18, VPS39, and VPS41. VPS18 and VPS11 build the core to which the SNARE-binding molecules, Sec1/Muinc18-like VPS33 and VPS16, are attached. Additionally, the HOPS complex has two Rab-interacting subunits, VPS41 interacting with Arf-like Arl8b protein and VPS39 interacting with Rab2^[98]^. The HOPS complex stabilizes the docking of autophagosomes to lysosomes and promotes SNARE complex assembly, facilitating membrane fusion. Structural studies have revealed that HOPS serves as a physical and functional scaffold during the STX17-mediated fusion process. VPS33A, as a subunit of HOPS complex, binds to SNARE proteins like STX17 on opposing membranes, controlling the progression of membrane fusion between autophagosomes and lysosomes, thus determining the place where fusion occurs^[99]^. The recruitment to the STX17-positive autophagosomes is regulated by RUBCNL/PACER (rubicon like autophagy enhancer)^[99]^.

Mutations in specific HOPS subunits have been linked to neurodegenerative and lysosomal storage disorders. VPS41 mutations impair lysosome biogenesis and vesicle trafficking, leading to disorders marked by defective autophagic clearance^[100]^. Patients with autosomal recessive VPS41 mutations present with progressive developmental disorders, motor dysfunction including ataxia and dystonia, as well as abnormal membrane-bound vesicles in lymphocytes and lymphoblastoid cells^[101]^. Patients with VPS16 mutations present with early-onset oromandibular, bulbar, cervical or upper limb dystonia which slowly progresses to a generalized dystonia at adulthood^[102]^.

The lysosome is a cellular signaling hub at the point of convergence of endocytic and autophagic pathways, where defective cargo is degraded and recycled. Pleckstrin homology domain-containing family member 1 (PLEKHM1) acts as an adaptor to facilitate the fusion of endocytic and autophagic vesicles with the lysosome. PLEKHM1 is a Rab7 effector protein interacting directly with the HOPS complex through the subunits VPS39 and VPS41, and localizes to vesicle contact sites such as autophagosomes where PLEKHM1 is recruited via interaction with ATG8/LC3. Defects in PLEKHM1 disrupt both endocytic and autophagic pathways^[102]^. While the cellular consequences of this disruption appear to be cell type-dependent, PLEKHM1 dysfunction has been shown to promote the accumulation of defective ribosomal products and impair the clearance of puromycin-induced aggresome-like induced structures (ALIS)^[103]^. As a potential clinical clue as to the effects of PLEKHM1 dysfunction, a recent GWAS study for Parkinson’s disease has reported reduced PLEKHM1 mRNA expression in the cerebellum of PD patients^[104]^. TRIM22, a member of the TRIM (tripartite motif) protein family, has only recently been identified as a positive regulator of autophagy^[105]^. TRIM22 facilitates the autophagosome-lysosome fusion by mediating the association between PLEKHM1 and GABARAP, while the TRIM22 R321K mutation has been shown to disrupt this fusion process, ultimately impairing autophagic clearance of neurotoxic protein aggregates in early-onset familial Alzheimer’s disease^[106]^.

### EPG5: key tethering factor

EPG5 (ectopic p-granules autophagy protein homolog 5) is a tethering factor that bridges autophagosomes and lysosomes by interacting with Rab7 and lysosomal surface proteins, such as LAMP2 and VAMP7. Additionally, EPG5 collaborates with WDR45, a component critical for autophagosome maturation and lipid metabolism. WDR45 facilitates the lipidation of LC3, indirectly supporting the recruitment of EPG5 to the autophagosomal membrane. These interactions ensure the precise alignment and docking of vesicle membranes, creating a platform for SNARE complex assembly and subsequent fusion. EPG5 also stabilizes the interaction between the HOPS complex and autophagosomes, enhancing the efficiency of the tethering process. Additionally, it stimulates the formation and stabilization of the STX17-SNAP29-VAMP8 SNARE complex^[16]^.

Following the first attribution of the autophagy disorder Vici syndrome to biallelic variants in the ectopic P-granules 5 autophagy tethering factor gene (*EPG5*)^[107]^, recessive mutations in EPG5 have been connected to a wide spectrum of multi-system phenotypes that are all driven by failure of autophagosome-lysosome fusion, resulting in impaired cargo delivery to the lysosome^[108]^. Loss-of-function mutations in EPG5 have been extensively studied, with a recent study by our group and others identifying their role in defective autophagic clearance^[43,108,109]^. The phenotypical spectrum ranges from aberrant neurodevelopment to neurodegeneration and seizures, mimicking features of human ALS in an *Epg5* mouse model^[108]^. Classic Vici syndrome (VS) represents the more severe end of the spectrum, due to a homozygous or compound heterozygous allelic state for at least one truncating variant resulting in reduced EPG5 protein expression, indicating a critical threshold for EPG5 protein levels in disease manifestations. Patients with classic VS show callosal agenesis, microcephaly, cataracts, cardiomyppathy, immunodeficiency, hypopigmentation, and failure to thrive^[110]^. The more general term *EPG5*-related disorder has been recently introduced to also include patients with mostly neurodevelopmental disorders that do not show the full clinical picture of VS. Genome sequencing in such patients showed homozygosity or compound heterozygosity for *EPG5* missense variants, leading to a presumably dysfunctional but not necessarily substantially reduced EPG5 proteins in these patients, corresponding to the relatively more subtle clinical symptoms^[42]^.

Recent findings by our group have further elaborated on the various EPG5 functions, showing that EPG5 mutations also interfere with the recruitment of critical fusion proteins, such as RAB7, compounding deficits in vesicle tethering and fusion^[42]^. Furthermore, *EPG5*-mutant cells exhibit compromised lysosomal enzyme delivery, exacerbating systemic phenotypes of impaired autophagy and neurodegeneration. We have linked pathogenic *EPG5* variants to severe neurodevelopmental and systemic phenotypes, including impaired immune responses and progressive neurodegeneration^[42]^.

### RUBCN: a negative regulator

RUBCN (RUN Domain Beclin-1-Interacting And Cysteine-Rich Domain-Containing Protein) is one of the few negative regulators of autophagy^[111]^. It inhibits autophagosome-lysosome fusion by interacting with RAB7 with its Rubicon homology domain (RH domain) and by sequestering UVRAG (UV radiation resistance associated protein), which promotes autophagy by activating the PI3P complex, via its phosphatidylinositol kinase binding domain (PIKB domain) to inhibit the HOPS complex^[112,113]^. Additionally, RUBCN inhibits autophagosome maturation by inhibiting phosphatidylinositol 3-kinase (PIK3C), a subunit of PI3K complex, function by interacting with VPS34 from HOPS with its N-terminal RUN domain^[112]^. RUBCN is also involved in non-canonical autophagic functions like ATG8/LC3-associated phagocytosis (LAP) and ATG8/LC3-associated endocytosis (LANDO)^[113]^.

A recent study showed RUBCN isoforms, a large 130kDa (RUBCN-130) and a smaller 100 kDa isoform (RUBCN-100) lacking the RUN domain, with distinct functions: RUBCN-130 is preferentially located to LAMP1-positive lysosomes, while RUBCN-100 is located to EEA-positive early endosomes. Selective RUBCN-130 deficiency enhances autophagy, humoral response, B_mem_ cell differentiation, and supresses mTORC1 activity, while RUBCN-100 increases VPS34 activity at early endosomes, which suggests that RUBCN-130 functions in autophagosome-lysosome fusion inhibition, and that RUBCN-100 plays a role in LAP and LANDO^[114]^.

The Rubicon homologue increases with age in worms, flies, and mice under physiological conditions, but its expression is decreased with caloric restriction and in several long-lived C. elegans mutants, in keeping with the observation that its genetic deletion causes life- and healthspan extension in worms and flies^[13]^. Furthermore, Rubicon deletion (of the 130kDa band, but not the slightly expressed 100kDa Band) prevented several age-associated phenotypes in mice, including decreased Lewy body and α-synuclein formation, a characteristic feature of Parkinson’s disease^[13]^. A recent study investigated the TANK binding dinase 1 (TBK1) dependent RAB7 phosphorylation of Ser72 which leads to a differential binding of either RUBCN (unphosphorylated) or Pacer (phosphorylated)^[79]^. While RUBCN inhibited autophagosome-lysosome fusion, the binding of Pacer to RAB7 was needed for Parkin-dependent mitophagy^[79]^. Interestingly, this TBK1 phosphorylation of RAB7 is increased in amino acid-enhanced conditions and relieves RAB7 inhibition of the mTORC1 complex^[115]^. The same study also described a TBK1 pathogenic variant (E696K) associated with ALS-FTD, that constitutionally accumulates at lysosomes and leads to increased RAB7 pSer72 phosphorylation and increased mTORC1 activity^[115]^. These observations indicate that Parkin-dependent mitophagy occurs in amino-acid-rich conditions with simultaneous activation of the mTORC1 complex.

A rare mutation in the RH domain of RUBCN leads to reduced colocalization of RAB7 and RUBCN, leading to Salih ataxia, a disease that causes epilepsy, delayed walking and gait ataxia, upper and lower limb movement disorders, dysarthria, diminished upper limb tendon reflexes, nystagmus or saccadic eye pursuit, and mild cerebellar vermis atrophy with dilated interfoliar sulci at 18 years of age^[116,117]^.

While the inhibitory role in canonical autophagy seems to exacerbate neurodegenerative diseases, RUBCN’s non-canonical role in LANDO seems to have a protective role in Alzheimer’s disease by clearing extracellular Amyloid β plaques^[118,119]^. Additionally, RUBCN is induced by EGFR and RUBCN interaction with Beclin-1 is enhanced by EGFR-dependent phosphorylation of Beclin-1, which inhibits autophagy and exacerbates atherosclerosis, cancer, and type 2 diabetic nephropathy, but also plays a crucial role in phagocytosis of photoreceptor outer segments by macrophages and retinal pigment epithel cells via LAP^[120–123]^. In contrast, in proximal tubular epithelial cells (PTECs), RUBCN knockout leads to cellular membrane transport to lysosomes and causes metabolic syndrome^[124]^. RUBCN also plays an important role in nonalcoholic fatty liver disease, where it impairs autophagic flux, increases lipotoxicity, and hepatocyte-specific knockout of RUBCN in mice on a high-fat diet improves liver steatosis, injury, and attenuates endoplasmatic reticulum-stress and autophagy impairment^[125]^. In cholestasis, chenodeoxycholic acid is the most commonly retained bile acid species and the most potent farnesoid X receptor (FXR) agonist^[126]^, leading to direct FXR induction of RUBCN and impaired autophagy^[126]^. Ursodesoxycholic acid is a FXR antagonist and an established treatment for several liver pathologies^[126]^. In adipocytes, RUBCN mediates the fasting response and adipocyte-specific knockout exhibits systemic fat loss^[127]^. RUBCN also plays an important role in viral immune response, where conflicting results regarding its function have been reported: Firstly, RUBCN has been shown to inhibit interferon production by interacting with NF-κB essential modulator (NEMO), a key factor in the IFN pathway^[128]^. A later study investigating Hepatitis C virus (HCV) described increased signaling in the type I IFN pathway, when late-stage, but not early-stage autophagy is inhibited by RUBCN overexpression, Bafilomycin A1 (an Inhibitor of the V-ATPase leading lysosomal dysfunction), or chloroquine which inhibited HCV replication^[129]^. SARS-CoV 2 and HIV-1 also use proteins similar to RUBCN to inhibit autophagosome-lysosome fusion (ORF3a in SARS-CoV 2 and Nef in HIV-1)^[130,131]^. The RUBCN-130 starting exon is also alternatively spliced and a recent study mapping RNA isoform diversity in the human frontal cortex of healthy and Alzheimer’s disease patients reveals RUBCN-130 increase and RUBCN-100 decrease in patients^[132,133]^. This makes the specific deletion of RUBCN-130 an attractive target for enhancing autophagosome-lysosome fusion without affecting the role of RUBCN in non-canonical autophagy pathways.

## Key changes of the autophagosome-lysosome fusion machinery in physiological aging

Aging represents a continuum of cellular and systemic decline driven by accumulating damage on a cellular and tissue level. While autophagosome-lysosome fusion dysfunction is not the sole driver of aging-related pathology, it can act as both a contributor and amplifier of cellular damage. The interplay between defective fusion, proteostasis imbalance, oxidative stress, and inflammation form a vicious circle, exacerbating age-related diseases and frailty^[134]^.

Aging is characterized by a suppression in autophagic activity and efficiency^[13]^. The overexpression or hyperactivation of autophagy inhibitors in neurons, most prominently the negative regulator *RUBCN*, contributes significantly to the pathological cascades underlying neurodegeneration^[10]^. By obstructing key stages of autophagic flux, these negative regulators allow toxic substrates to accumulate, accelerating cellular damage and neuronal loss. Their influence highlights the importance of maintaining a delicate balance in autophagic regulation for neuronal health and resilience against age-related and disease-associated stressors.

Recent evidence has shown a general increase and enhanced enzymatic activity in PINK1-PRKN, both important mitophagy regulators, under basal conditions *in vivo* in different energetically demanding tissues of mice during normal aging^[135]^. The most pronounced activation and flux were in mouse heart and less pronounced in brain or skeletal muscle, raising the question of a differential effect of autophagy in organ-specific aging processes.

Considering autophagosome-lysosome fusion on a cellular and tissue level, the most prominent features in biological aging are dysregulations in autophagic flux, lysosomal integrity failure, and chronic inflammation. Any such manifestations of biological aging are also found in neurodegenerative diseases. Table 2 demonstrates similarities of findings in cellular models of aging and neurodegenerative disorders, with the exacerbation potential of aging-related changes highlighted for the latter.

**Table 2.** Molecular biological findings in aging and neurodegenerative diseases are divided into three exemplary categories: autophagic flux, lysosomal integrity, and chronic inflammation.

Feature	Aging	Disease
Autophagic flux		
Autophagic vacuole accumulation	RAB7 activity diminishes, HOPS complex stability is reduced, and RUBCN expression increases^[10]^ contributing to the accumulation of autophagic vesicles^[13]^	Associated with AD, PD and metabolic diseases^[10,79,136]^
Tethering impairment	Roles of EPG5 and WDR45 during healthy aging are yet unclear	Early dementia and parkinsonism with EPG5 and WDR45 defects^[42,137]^
Lysosomal integrity		
Lipofuscin burden	Accumulation of lipofuscin interferes with lysosomal proteolysis^[138]^	Reduced efficiency of autophagosome clearance, creating a feedback loop that further accelerates lysosomal damage
Cathepsin leakage	Lysosomal membrane permeability increases with age, leading to the release of cathepsins into the cytoplasm.	Triggers caspase-independent apoptosis, contributing to neuronal loss in diseases like AD and PD
Chronic inflammation (‘inflammaging’)		
Damage-associated molecular patterns (DAMPs)	Lysosomal rupture releases damage-associated molecular patterns (DAMPs), such as mitochondrial DNA and oxidized lipids^[139]^	DAMP release activates inflammatory pathways (NLRP3 inflammasome and cGAS-STING) in mitochondria, leading to neurodegenerative diseases^[140]^
Senescence-Associated Secretory Phenotype (SASP)	Fusion defects contribute to the senescence of aging cells, which release pro-inflammatory cytokines	Perpetuating systemic inflammation promotes astrogliosis in AD^[141]^

## Role of autophagy and the autophagosome-lysosome fusion machinery in more common neurodegenerative diseases

### Alzheimer’s disease (AD)

Autophagic vesicles may accumulate within dystrophic neurites in Alzheimer’s Disease (AD), reflecting a blockade in autophagic flux. Defective RAB7 and HOPS activity in AD impairs amyloid-beta and tau clearance, leading to the accumulation of plaques and tangles^[142,143]^. Emerging evidence suggests that lysosomal dysfunction precedes amyloid-beta deposition, with reduced acidification and proteolytic capacity exacerbating amyloidogenic and tau pathology^[144]^. EPG5 dysfunction has been implicated in AD pathology with several single nucleotide polymorphisms (SNPs)^[145]^. Furthermore, RUBCN has been shown to play a critical role in AD progression, by demonstrating that RUBCN knockout in mice results in an intensified amyloid β burden in the hippocampus and decreased Pacer and p62 levels^[136]^, highlighting RUBCN as a significant contributor to neurodegeneration in AD. Altered SNARE protein function in AD further impairs the autophagosome-lysosome fusion process, amplifying neuronal vulnerability^[146]^. In AD, elevated levels of negative regulators, particularly Rubicon and mTOR, create significant bottlenecks in autophagic clearance^[136]^. Rubicon inhibits the autophagosome-lysosome fusion step, leading to the accumulation of amyloid-beta (Aβ) and tau-containing vesicles^[136]^. These aggregates not only cause proteotoxicity but also impair synaptic transmission and plasticity, which are critical for memory and learning. Additionally, mTOR hyperactivation blocks autophagy initiation, preventing the removal of misfolded proteins and exacerbating endosomal-lysosomal dysfunction, which is a hallmark of AD pathology^[147–149]^.

### Parkinson’s disease (PD)

The onset of parkinsonism due to monogenically determined autophagic dysregulation can vary from infantile dystonia-parkinsonism to juvenile dystonia-parkinsonism (e.g., *EPG5*- or *WDR45*-related disorders^[42,137]^), early adult parkinsonism and late adult parkinsonism (e.g., *PINK1*- or *PRKN*-related disorders^[150]^). Of note, pathogenic variants in *GBA* have been reported with childhood parkinsonism in biallelic states while relatives of patients had adult onset parkinsonism in monoallelic states^[151,152]^.

Impaired mitophagy, due to dysregulated RAB7 or HOPS function, results in the persistence of damaged mitochondria, increased oxidative stress, and α-synuclein aggregation. Dopaminergic cell death in Parkinson’s disease is associated with the accumulation of protein aggregates called Lewy bodies (LBs) containing vesicular membrane structures and organelles in conjunction with α-synuclein (α-Syn). Overexpression of α-Syn led to stalled autophagosome-lysosome fusion through abundance of SNAP29^[153]^. *SNAP29* knockdown mimicked the effect of α-Syn on autophagy, whereas SNAP29 co-expression reversed the α-Syn-induced changes on autophagy and ameliorated dopaminergic neuronal cell death^[153]^.

In PD, the failure of mitophagy due to Rubicon overexpression leads to the persistence of dysfunctional mitochondria which are major sources of oxidative stress. This exacerbates α-synuclein aggregation and the subsequent formation of Lewy bodies, key pathological features of PD^[154]^. Furthermore, Rab7 inactivation caused by Rubicon impairs vesicular trafficking and lysosome dynamics, which are critical for dopaminergic neuron survival. BCL-2’s interaction with Beclin-1 also limits autophagy induction, reducing the degradation of protein aggregates and damaged organelles^[155]^.

Recent studies highlight the role of lysosomal enzyme deficiencies, such as glucocerebrosidase mutations, in further disrupting autophagosome maturation and clearance. EPG5 dysfunction has also been identified as a contributing factor in PD, through *EPG5*-related impairment of autophagosome tethering and PINK1/Parkin-dependent mitophagy, also accelerating dopaminergic neuron loss^[42]^. RUBCN has also been implicated in the modulation of Parkin-mediated mitophagy. RUBCN inhibits autophagosome-lysosome fusion by directly interacting with RAB7 and negatively regulates the recruitment of the HOPS complex to autophagosomes^[79]^. This interference delays the clearance of damaged mitochondria, exacerbating oxidative stress and neuronal damage. The spectrum of RUBCN’s impact ranges from early-onset motor disorders, such as hereditary spastic paraplegia, to late-onset adolescent or adult-onset motor disorders such as Parkinson’s disease. RUBCN overexpression contributes to progressive motor deficits by impairing mitophagy across these disorders. This mitochondrial and lysosomal crosstalk dysfunction underscores the shared pathological features and continuum of movement-related neurodegenerative diseases.

### Amyotrophic lateral sclerosis and frontotemporal dementia (ALS-FTD)

Possibly the strongest connection between autophagy and a common age-related neurodegenerative disease is with the ALS-FTD spectrum^[156]^. Several genes mutated in ALS-FTD regulate autophagy. Some of these genes specifically affect the autophagosome-lysosome fusion and digestion. For instance, progranulin (PGRN) homozygous null mice showed potential dysfunction at the level of autophagosome clearance, and an accumulation of undigested LC3-positive autophagosomes was also observed *in vivo*^[157]^. Valosin-containing protein (VCP) knockdown in *Drosophila melanogaster* resulted in a shift from Atg8-positive vesicle colocalisation with the lysosome network to a collapsed lysosome network with adjacent Atg8 positive vesicles, indicating blocked fusion^[158]^. CHMP2B mutations cause FTD and also ALS and evidence indicate that autophagy function is impacted at the late endosome and lysosome fusion stages, likely a consequence of CHMP2B function in the ESCRT-III complex^[159]^. Finally also C9ORF72, the most common cause of ALS-FTD, has been reported to have a role at the lysosomal stage which may contribute to the C9-ALS-FTD pathology^[160]^. In addition to this, autophagic clearance intersects with protein accumulation and aggregation of TDP-43, FUS, SOD1 and the C9ORF72-generated DiPeptide Repeats (DPRs) following the blueprint of the mutual interference between proteotoxic species and autophagy observed in all neurodegenerative diseases associated with defective proteostasis^[156]^.

### PolyQ diseases

Polyglutamine (PolyQ) disorders, including Huntington’s disease (HD) and dentatorubral-pallidoluysian atrophy (DRPLA), are characterized by the accumulation of misfolded proteins and defective autophagy^[161]^. While in HD the early steps of autophagy initiation and nucleations appear to be primarily affected^[162,163]^, the final steps of fusion and digestion are specifically impaired in DRPLA, leading to the accumulation of undigested auto-lysosomes and cell degeneration^[164–166]^. Emerging studies also highlight the importance of nucleophagy, the selective autophagic removal of damaged nuclear material, in DRPLA^[166,167]^. Inhibition of autophagic clearance causes nuclear degeneration and a novel form of cell death, karyoptosis^[166]^. The specific role of nuclear polyQ aggregates in damaging the nuclear envelope has been recently reported with critical consequences for cell functioning^[168]^. Intriguingly, interfering with the fusion machinery, either by Bafilomycin A1 treatment of by STX17 or EPG5 knockdown causes nuclear degeneration and karyoptosis^[166]^. These findings underscore the importance of the fusion machinery and of nuclear autophagy in the progression of PolyQ disorders.

## Therapeutic strategies

Clinical trials directly modulating autophagosome-lysosome fusion are limited, but there is active preclinical research focused on modulating the fusion process to restore or enhance autophagic flux. Enhancing autophagy by sole mTOR inhibition through rapamycin or rapalogs has been demonstrated to a variable degree to protect from neurodegenerative diseases such as Alzheimer’s disease^[169–171]^ but is not expected to alleviate autophagosome-lysosome fusion disorders as it may further aggrevate the accumulation of toxic substances before the part of the pathway that is blocked^[2,134]^. Restoring lysosomal functionality through lysosomal acidification enhancers may prove a plausible strategy to mitigate the accumulation of toxic cellular wastes in autophagosomes perhaps via TFEB modulation^[172]^.

In the context of Parkinson’s disease, α-synuclein (α-syn) aggregation, considered as a biomarker of extensive neurodegeneration, may be exacerbated by impaired autophagy as Rab7 induction has been shown to mitigate α-synuclein accumulation and associated neurotoxicity^[173]^. Feasible approaches should not only aim to improve autophagic flux but also address the systemic implications of fusion impairment in aging and neurodegenerative diseases:

1. Enhancing RAB7 activity with small molecules that activate RAB7 or stabilize its GTP-bound state could restore fusion efficiency. Previous reports have focussed on restoration of autophagosome-lysosome fusion with the overactivation of (GTP-bound) Rab7 or its interacting proteins to reduce lysosomal and mitochondrial dysfunction and decrease neuronal apoptosis^[174,175]^. Several studies suggest that either phosphorylation at RAB7 serin 72 residue or modulation of Rab7 through PIPKIγi5 could serve as potential strategies to enhance RAB7 activity^[176,177]^. These modulations may enhance lysosomal function and support RAB7 activity, but potential compounds have to date only been assessed in preclinical models and to the best of our knowledge are currently not under development for clinical use^[178,179]^. Additionally, the widely used anti-diabetic medication metformin is an AMPK modulator that has been shown to improve autophagic flux and may enhance RAB7 activity^[180,181]^. These modulators hold promise in models of AD and PD, where impaired RAB7 function may at least partly contribute to autophagic deficits.
2. Targeting RUBCN strategies to inhibit its isoform-specific expression or function may ameliorate its inhibitory effects on autophagic flux. Genetic knockdowns of RUBCN in mice and worms have been demonstrated to enhance autophagic flux, diminish pathological protein accumulation such as α-synuclein-accumulation in the brain of mice, and alleviate age-related phenotypes, particularly when targeted in neuronal cells^[182]^. Inhibition of Rubicon can be achieved either by disrupting its interaction with key autophagy regulators such as Rab7 or Beclin1, or by modulating upstream effectors like Rock1, which controls Rubicon’s autophagy-suppressive activity and has been investigated in the context of acute ischemic stroke^[183]^. RNA interference and small-molecule inhibitors of RUBCN are being explored as therapeutic avenues.
3. Modulating SNARE assembly by enhancing SNARE protein function or stability using pharmacological chaperones could improve fusion in diseases that are characterized by impaired SNARE-mediated membrane fusion. Interestingly, the antiepileptic medication levetiracetam has been shown to modulate SNARE protein assembly and stabilize synaptic vesicle dynamics^[184]^. Although primarily studied in the context of synaptic function, its effects on SNARE complex stabilization suggest potential therapeutic relevance in diseases involving impaired autophagosome-lysosome fusion. There is preliminary evidence for levetiracetam’s modulation of vesicle fusion pathways, which may add to its potential in regulating autophagic flux in neuronal tissue^[184]^. Along similar lines, we have recently shown that Snap-25 knockdown in Epg5-deficient flies may reduce seizure frequency, further advancing the idea of SNARE complex modulation as a potential therapeutic strategy^[43]^. Regulatory processes, including post-translational modifications of SNARE proteins, especially the phosphorylation of VAMP8, are currently under investigation as potential therapeutic targets. Phosphorylation of VAMP8 has been shown to inhibit lysosomal fusion under basal conditions, while concurrently priming lysosomes for rapid fusion upon stimulation. This dynamic regulation suggests that therapeutic modulation of VAMP8 may offer a strategy to restore impaired autophagic flux in Alzheimer’s disease^[185]^. Organoids and genetically engineered animal models may provide physiologically relevant systems to study the fusion machinery and validate therapeutic strategies *in vivo* ^[186]^.

The use of any small-molecule therapy such as rapamycin or inducers of autophagosome-lysosome fusion in neurodegenerative diseases should also be considered in terms of blood-brain barrier permeability. The use of antisense oligonucleotides has been recently discussed for both oral and intrathecal administration, with the need for repetitive intrathecal administration through lumbar punctures potentially limiting its clinical utility^[187]^.

Preclinical studies may yield some potential therapeutic strategies in neurodegenerative diseases with adult onset but are largely limited in efficacy. This may arise from cohorting neurodegenerative diseases based solely on phenotypic presentation such as PD or AD. These clinical entities have largely become umbrella terms for a spectrum of monogenic disorders, some with clear delineations such as *PINK1*- or *PRKN*-related PD, while other perhaps more common monogenic disorders may yet remain unclear and subsumed as “idiopathic.” Wide genomic screening efforts in large cohorts have yielded several new disease genes for dementia or parkinsonism in adolescence or young adulthood, mostly in biallelic states including *EPG5*, *LYST*, and *RAB32*^[42,188,189]^. It is unclear whether any autophagy genes may impact dementia or parkinsonism at later stages of adulthood in a monoallelic state. While modulating autophagy in neurodegeneration has largely been researched in specific monogenic disorders, its translation to clinical use has been impeded by sampling neurodegenerative disease cohorts based on phenotypes instead of genotypes. We postulate that a more structured genotype-first approach in specific disease groups may in turn boost the efficacy of targeted therapy options, and benefit both basic scientific understanding of neurodegenerative causes and clinical application in neurodegenerative diseases.

## Conclusion

Autophagosome-lysosome fusion is a critical process for maintaining cellular homeostasis. Its dysregulation underlies a wide range of pathological states, from aberrant neurodevelopment to neurodegeneration and physiological aging. Advances in understanding the molecular mechanisms and pathophysiological implications of autophagosome-lysosome fusion defects have opened new avenues for therapeutic intervention. Continued research into this dynamic process will provide valuable insights into cellular resilience and vulnerability, potentially paving the way for innovative treatments focussing on autophagy-related diseases.

The decline in autophagosome-lysosome fusion efficiency with age is a critical driver of cellular and systemic aging. By impairing proteostasis, mitochondrial quality control, lysosomal integrity, and inflammation resolution, fusion dysfunction amplifies aging phenotypes and predisposes individuals to chronic diseases. A deeper understanding of these processes will provide crucial insights into the biology of aging and its associated pathologies.

## Data Availability

Data sharing is not applicable to this article as no data were created or analyzed in this study.
